# The Effect of Zn-Substitution on the Morphological, Magnetic, Cytotoxic, and In Vitro Hyperthermia Properties of Polyhedral Ferrite Magnetic Nanoparticles

**DOI:** 10.3390/pharmaceutics13122148

**Published:** 2021-12-14

**Authors:** Ionel Fizesan, Cristian Iacovita, Anca Pop, Bela Kiss, Roxana Dudric, Rares Stiufiuc, Constantin Mihai Lucaciu, Felicia Loghin

**Affiliations:** 1 Department of Toxicology, Faculty of Pharmacy, “Iuliu Hațieganu” University of Medicine and Pharmacy, Pasteur 6A, 400349 Cluj-Napoca, Romania; ionel.fizesan@umfcluj.ro (I.F.); anca.pop@umfcluj.com (A.P.); kbela@umfcluj.ro (B.K.); floghin@umfcluj.ro (F.L.); 2 Department of Pharmaceutical Physics-Biophysics, Faculty of Pharmacy, “Iuliu Hatieganu” University of Medicine and Pharmacy, Pasteur 6, 400349 Cluj-Napoca, Romania; cristian.iacovita@umfcluj.ro; 3 Faculty of Physics, “Babes Bolyai” University, Kogalniceanu 1, 400084 Cluj-Napoca, Romania; roxana.pacurariu@phys.ubbcluj.ro; 4 Department of Bionanoscopy, MedFuture Research Center for Advanced Medicine, “Iuliu Hatieganu” University of Medicine and Pharmacy, Pasteur 4-6, 400337 Cluj-Napoca, Romania

**Keywords:** zinc doped iron oxide magnetic nanoparticles, polyol method, specific absorption rate, in vitro magnetic hyperthermia, cellular uptake, cytocompatibility, A549 cells, BJ cells

## Abstract

The clinical translation of magnetic hyperthermia (MH) needs magnetic nanoparticles (MNPs) with enhanced heating properties and good biocompatibility. Many studies were devoted lately to the increase in the heating power of iron oxide MNPs by doping the magnetite structure with divalent cations. A series of MNPs with variable Zn/Fe molar ratios (between 1/10 and 1/1) were synthesized by using a high-temperature polyol method, and their physical properties were studied with different techniques (Transmission Electron Microscopy, X-ray diffraction, Fourier Transform Infrared Spectroscopy). At low Zn doping (Zn/Fe ratio 1/10), a significant increase in the saturation magnetization (90 e.m.u./g as compared to 83 e.m.u./g for their undoped counterparts) was obtained. The MNPs’ hyperthermia properties were assessed in alternating magnetic fields up to 65 kA/m at a frequency of 355 kHz, revealing specific absorption rates of up to 820 W/g. The Zn ferrite MNPs showed good biocompatibility against two cell lines (A549 cancer cell line and BJ normal cell line) with a drop of only 40% in the viability at the highest dose used (500 μg/cm^2^). Cellular uptake experiments revealed that the MNPs enter the cells in a dose-dependent manner with an almost 50% higher capacity of cancer cells to accommodate the MNPs. In vitro hyperthermia data performed on both cell lines indicate that the cancer cells are more sensitive to MH treatment with a 90% drop in viability after 30 min of MH treatment at 30 kA/m for a dose of 250 μg/cm^2^. Overall, our data indicate that Zn doping of iron oxide MNPs could be a reliable method to increase their hyperthermia efficiency in cancer cells.

## 1. Introduction

The most commonly used iron oxide magnetic nanoparticles (MNPs) for biomedical applications are magnetite-based (Fe_3_O_4_) due to their good biocompatibility, chemical stability, low toxicity profile, and superior biodegradability [1,2,3,4]. Under the influence of a high-frequency alternating magnetic field (AMF), MNPs generate heat due to their dynamic hysteresis response, which depends on their static hysteresis characteristics and on their ability to follow the changes in the AMF through relaxation mechanisms (as N éel and Brown) [5,6,7]. In this sense, numerous studies concerning magnetic hyperthermia (MH) have been centered on the development of Fe_3_O_4_ particles, exhibiting different sizes, shapes, and morphologies being either in a superparamagnetic (SP) or ferromagnetic (F) state at room temperature (RT), with the final aim of improving the therapeutic efficiency [8,9,10,11,12]. As proven by both in vitro and in vivo studies, the mobility of SP-Fe_3_O_4_ particles, responsible for Brownian relaxation, is inhibited when they are internalized into tumor cells, causing a decrease of the heating efficiency because the SP-Fe_3_O_4_ particles can only undergo Néel relaxation. Moreover, in the case of F-Fe_3_O_4_ particles, the intracellular clustering leads to a decrease in their heating efficiency as well [13].

In these conditions, the modification of the chemical composition of Fe_3_O_4_ MNPs represents an efficient strategy to improve their effectiveness in MH [9,14,15]. Magnetite has the capabilities to accommodate their inverse cubic spinel structure divalent transition metal ions, as cobalt (Co), manganese (Mn), zinc (Zn), and nickel (Ni) give rise to spinel-structured ferrites [16]. The equal distribution of trivalent Fe ions (Fe^3+^) in the two sublattices—tetrahedral (A) and octahedral (B)—of the spinel structure following an antiparallel magnetic configuration to an applied magnetic field leads to the cancelation of their magnetic moment. Thus, the net magnetic moment of the Fe_3_O_4_ particles is given by the divalent iron ions (Fe^2+^) located in the B sites, which is 4μ_B_ (Bohr magneton) per formula unit. In this context, the substitution of Fe^2+^ (d^6^) cations in B sites with Mn^2+^ (d^5^) cations, bearing a larger magnetic moment of 5μ_B_ per formula unit due to their five unpaired valence electrons, led to an increase of the saturation magnetization (M_s_) of ferrite particles and thus to an enhancement of their heating capabilities [17,18,19,20,21]. The replacement of Fe^2+^ (d^6^) cations in B sites by more anisotropic Co^2+^ (d^7^) cations, conducted to ferrite particles with larger coercivity values and a wider hysteresis loop, which improved considerably the production of heat under AMF [22,23,24,25]. A particular different situation is offered by the diamagnetic Zn^2+^ (d^10^) cations once incorporated in the spinel structure, as they can produce significant enhancement of the ferrite particle′s magnetic moment, compared to pure Fe_3_O_4_ [26,27,28]. This is due to the unique tendency of Zn^2+^ cations to occupy the A sites in the spinel structure, forcing the trivalent Fe^3+^ (d^5^) cations to migrate to B sites by replacing the divalent Fe^2+^ cations. This scenario holds up until a certain Zn content in the spinel structure, which depends on the employed synthetic route. Above this Zn doping threshold, the lack of magnetic moments at A sites disrupts the exchange interaction between both sublattices, producing a decrease in the net magnetic moment of ferrite particles.

From this point of view, the Zn ferrites (ZnF) particles hold great promise for both in vitro and in vivo MH experiments, as the high increase of their M_s_ will cause an enhancement of the heat dissipation by Néel relaxation once immobilized inside cells/tumors. In addition, it has been shown that the ZnF particles are stable against oxidation, being presumably biocompatible since Zn displays a relatively high toxic dose (450 mg/day) in the human body [29,30]. Several synthesis routes have been employed for the preparation of ZnF particles, exhibiting different particle size histograms, cation distribution between the two sublattices, and magnetic properties. The preparation of single-crystalline monodisperse ZnF-particles with magnetic properties closed to bulk-like ones has been achieved by thermal decomposition of metal acetylacetonates and oleates in organic solvents at high temperatures [25,26,28,29,31,32,33,34]. In most cases, this method enables the formation of secondary phases as wüstite [35]. Therefore, the most popular approach utilized co-precipitation of metal chloride or nitrate salts as it is quick, simple, and with low costs [27,36,37,38,39,40,41]. The resulting ZnF-particles are, in the majority of cases, polydisperse in size and shape. In addition, other synthesis methods such as microwave-assisted [42], hydrothermal [43], polyol [44,45], or biosynthesis [46] have been used.

Despite the previous studies, there is still plenty of room to develop synthetic routes for the preparation of ZnF-particles. We have recently shown that the polyol method performed at elevated temperature (300 °C) and high pressure enables the formation of polyhedral Fe_3_O_4_ particles with enhanced magnetic and hyperthermia properties [47]. Therefore, herein, we explored this modified polyol method to elaborate ZnF-particles. By finely modifying the Zn/Fe molar ratio in the synthesis conditions, a series of ZnF particles of different sizes, shapes, and zinc contents have been obtained. These ZnF samples have been morphologically, structurally, and magnetically analyzed with great accuracy making use of TEM, XRD, FT-IR spectroscopy, and DC magnetometry. The MH performances of ZnF particles were assessed in water at two different iron concentrations at a fixed frequency (355 kHz) and variable AMF strengths (5–65 kA/m). Two types of ZnF (ZnF01 and ZnF02) particles providing the best heating performances were further selected to evaluate their cytotoxicity, cellular uptake, and in vitro MH performance on one cancer cell line—human pulmonary cancer cells (A549), and one normal cell line—human foreskin fibroblasts (BJ). The cytotoxicity and intracellular MH were evaluated by using two complementary assays: the neutral red (NR) uptake assay, and the Alamar Blue (AB) assay, while the cellular uptake of ZnF01 and ZnF02 particles was evaluated qualitatively and quantitatively after a 24 h incubation. The MH performance in selectively inducing cellular death in cancerous cells at three different field intensities (30 kA/m, 45 kA/m, 60 kA/m) and a constant frequency (355 kHz) of the AMF was evaluated as well.

## 2. Materials and Methods

### 2.1. Materials

All the reagents used in the current study were of analytical grade and were used without any additional purification. The ZnF particles were obtained by using the following products: iron (III) chloride hexahydrate (FeCl_3_·6H_2_O) (Carl-Roth, Karlsruhe, Germany, ≥98%), zinc (II) chloride hydrate (ZnCl_2_) (Carl-Roth, Karlsruhe, Germany, ≥99.5%), sodium acetate (NaAc) (Carl-Roth, Karlsruhe, Germany, ≥99.5%), and polyethylene glycol 200 (PEG200) (Carl-Roth, Karlsruhe, Germany, ≥99%).

### 2.2. Synthesis Method

The synthesis of ZnF particles was performed using a polyol mediated synthetic route, as follows: 1 mmol of FeCl_3_·6H_2_O, 25 mmol of NaAc, and a variable amount of ZnCl_2_ (0.1, 0.2, 0.4, 0.6, 0.8, and 1.0 mmol) were mixed and dissolved in 40 mL PEG200 in a 60 mL round-bottom flask. Upon magnetic stirring at 50 °C (500 rot/min for 30 min), the solutions were transferred in a homemade stainless steel reaction vessel, degassed by exposure to a flux of gaseous nitrogen for 5 min, and sealed using a Teflon gasket and five screws. The reaction vessel was introduced into an oven (Nabertherm GmbH, Lilienthal, Germany) equipped with a temperature controller (JUMO dTron 316, JUMO GmbH & Co., KG, Darmstadt, Germany) that allowed for programming the heating. The solutions were heated from RT to 300 °C with a constant heating rate of 3 °C/min and kept at this temperature for 1 h. After cooling the vessel at RT, the black precipitates were collected by neodymium magnet and the excess liquid was discharged. Five ultrasonication (15 min)/magnetic separation cycles using ethanol/double distilled water (v:v = 1:2) solvent were employed to remove the excess ligands and unreacted precursors. Finally, the black precipitates were kept in 10 mL double distilled water for further analysis.

### 2.3. Characterization Methods

The Transmission Electron Microscopy (TEM) samples were prepared by pipetting a 5 μL drop of the aqueous suspension of ZnF particles on carbon-coated copper grids, followed by the removal of the excess water and drying under ambient air. TEM analysis was performed on a Hitachi HT7700 (Hitachi Ltd., Tokyo, Japan) setup operating at 100 kV in high contrast mode. An 8-megapixel Charge-Coupled Device (CCD) camera was employed to capture the images.

X-ray diffraction (XRD) measurements were carried out using a Bruker D8 Advance diffractometer with Cu K radiation (Bruker AXS GmbH, Karlsruhe, Germany). The measurements were performed on powder samples obtained by drying the ZnF particles in a rota-evaporator. The FullProf software (FullProf.2k, -Version 7.00-May 2019-ILL JRC https://www.ill.eu/sites/fullprof/, accessed on 10 December 2021, Grenoble, France) was employed to detect the crystalline phases and to calculate the lattice parameters.

For Infrared-Spectroscopy (IR), ZnF particles were collected by a neodymium magnet, placed on the diamond crystal, and dried using a fan. The instrument used was a Bruker TENSOR II (Bruker Optics Inc., Billerica, MA, USA) in an attenuated total reflectance mode, using the platinum attenuated total reflectance (ATR) accessory with a single reflection diamond. The spectra were recorded in the 400–4000 cm^−1^ spectral range, at a resolution of 4 cm^−1^.

Hydrodynamic size measurements of ZnF samples were determined using a Zetasizer Nano ZS90 (Malvern Instruments, Worcestershire, UK) in a 90° configuration. The colloidal concentration of ZnF particles were 0.1 and 0.01 mg_MNPs_/mL.

Magnetic hysteresis loops were performed on powder samples, obtained by drying the ZnF particles in a rota-evaporator, at 300 K and under a range of external fields of ±4 T, using a Cryogenic Limited (London, UK) vibrating sample magnetometer (VSM).

For the magnetic hyperthermia measurements, ZnF particles suspended in 0.5 mL water at two different concentrations, were placed in the center of an 8-turn coil, made of a water-cooled copper tube, using a thermally isolated Teflon holder. Easy Heat 0224 from Ambrell (Scottsville, NY, USA), equipped with a fiber-optic thermometer and capable to deliver AC magnetic fields with variable amplitude (5–65 kA/m) at a frequency of 355 kHz, was used [48].

### 2.4. Cell Lines

One cancerous cell line (human pulmonary cancer cells—A549) and one normal cell line (human foreskin fibroblasts—BJ) were used as models in the present study. Both types of cells were purchased from American Type Culture Collection (ATCC, Manassas, VA, USA) and cultured in Dulbecco’s Modified Eagle Medium (DMEM, Gibco, Paisley, UK) supplemented with 10% Fetal Bovine Serum (FBS, Gibco, Paisley, UK). Cells were cultured in T75 flasks at a temperature of 37 °C in a humidified incubator with 5% CO_2_ supplementation, while the medium was changed every other day. At 80% confluency, cells were subcultured or used in experiments.

### 2.5. In Vitro Cytotoxicity Assays

For the cytocompatibility evaluation of the ZnF particles, two complementary assays, namely AB and NR assays, were performed on A549 and BJ cells after an exposure of 24 h. Similar to our previously published study, before the study of the nanomaterial’s cytocompatibility, the optical and biochemical interferences were evaluated [45]. For both assays, 750,000 A549 cells and 275,000 BJ cells were pre-plated for 24 h in 6 well plates to achieve a 70–80% confluency upon exposure to ZnF01 and ZnF02 particles. Cells were incubated for 24 h with 1 mL of increasing concentrations of particles suspensions to reach doses of 31.25, 62.5, 125, 250, and 500 µg/cm^2^. Following the exposure, cells were washed with phosphate-buffered saline (PBS, Gibco, Paisley, UK) and incubated with the AB and NR dyes. For the AB assay, cells were incubated with 2 mL of a 200 µM resazurin solution for 3 h, and the measurement of the fluorescence was performed at an λ_excitation_ = 530/25 nm; λ_emission_ = 590/35 nm. In the case of the NR assay, cells were incubated with a 40 μg/mL filtered NR dye solution for 2 h. Post-incubation, cells were washed to eliminate non-internalized dye and the accumulated cellular dye was extracted in a 50% hydroalcoholic solution containing 1% glacial acetic acid. The fluorescence was measured at a λ_excitation_ = 530/25 nm; λ_emission_ = 620/40 nm. Fluorescent measurements were performed using Synergy 2 Multi-Mode Microplate Reader. All experiments were done in triplicates and included a negative control (cells exposed to media) that was used for data normalization (100%).

### 2.6. Iron Concentration Determination

The iron content of samples was determined using the Liebig reaction. Briefly, 5 mg of ZnF particles were magnetically separated and further suspended in 10 mL of 12% HCl solution for digestion at 80 °C for 6 h. After the digestion step, the solutions were centrifuged at 12,000× *g* for 10 min and the supernatants were collected. For the iron (Fe^3+^) quantification, 50 µL of the supernatant were mixed for 30 min with 50 µL of 1% ammonium persulfate in 96 well plates to oxidize all iron content to Fe^3+^. Subsequently, 100 µL of 0.1 M potassium thiocyanate was pipetted—the absorbance of the red-colored iron-thiocyanate complex was measured at λ = 490 nm using Synergy 2 Multi-Mode Microplate Reader. The iron (Fe^3+^) present in the nanoparticles was calculated from a Fe^3+^ standard curve, with concentrations ranging between 7.8–250 µg/mL (Section S6 in Supplementary Materials).

### 2.7. Evaluation of Cellular Uptake

The cellular uptake of ZnF01 and ZnF02 particles were visualized using Prussian Blue staining, while the quantitative measurement was done by the Liebig reaction of free Fe^3+^ with thiocyanate, as previously described. For both assays, A549 and BJ cells were seeded for 24 h in 6-well plates at a density of 750,000 cells and 275,000 cells, respectively. Cells were exposed to ZnF01 and ZnF02 for 24 h at a concentration of 62.5, 125, and 250 µg/cm^2^. Following the exposure, cells were washed 2× with PBS, trypsinized, and centrifuged at 4500× *g* for 5 min, then further processed as described in Section 2.6 for the quantitative evaluation of the internalization. For the qualitative assessment, cells were fixed with a solution of 4% paraformaldehyde for 30 min at RT. The intracellular Fe^3+^ was stained for 30 min with a mixture containing a 2% HCl aqueous solution and a 2% potassium ferrocyanide (II) trihydrate aqueous solution (Prussian Blue stain). After the color development, cells were washed 3× with PBS and visualized under a light microscope. Representative images were taken at a magnification of 100×.

### 2.8. In Vitro Magnetic Hyperthermia

A549 and BJ cells seeded in 6-well plates were exposed for 24 h to 62.5, 125, and 250 µg/cm^2^ ZnF01 and ZnF02 nanoparticles. Cells were washed 2× with PBS to remove weakly bound particles and incubated with 300 µL of trypsin (0.05%) for 5 min. Trypsin was neutralized with 2700 µL of media and the cellular suspension was equally split in two aliquots that were further centrifuged for 10 min at 100× *g*. After removal of 1300 µL of supernatant, one aliquot was exposed to an AMF for 30 min, while the other aliquot maintained at 37 °C served as a negative control. For all the MH experiments, the frequency was set at 355 kHz and the intensities of the AMF studied were 30, 45, and 60 kA/m. After the AMF exposure, the cells, including those of the negative control, were seeded in 96-well plates as 6 technical replicates. Cellular viability was evaluated after 24 h using the AB and NR assays, and the results were expressed as relative values compared to the MH negative control (cells exposed to ZnF01/ZnF02, but not to the AMF). Each experiment was conducted with 3 biological replicates, each biological replicate including 6 technical replicates.

### 2.9. Statistics

The data are expressed as mean values ± standard deviation (SD). If not stated otherwise, the normally distributed data sets were analyzed using one-way analysis of variance (ANOVA) with a post-hoc + Dunn’s test. Data analyses and graphical representation were done using the SigmaPlot 11.0 computer software (Systat, Software Inc., Chicago, IL, USA). Results showing *p* values less than 0.05 were considered statistically significant.

## 3. Results and Discussion

### 3.1. Morphological Properties

**Figure 1:**
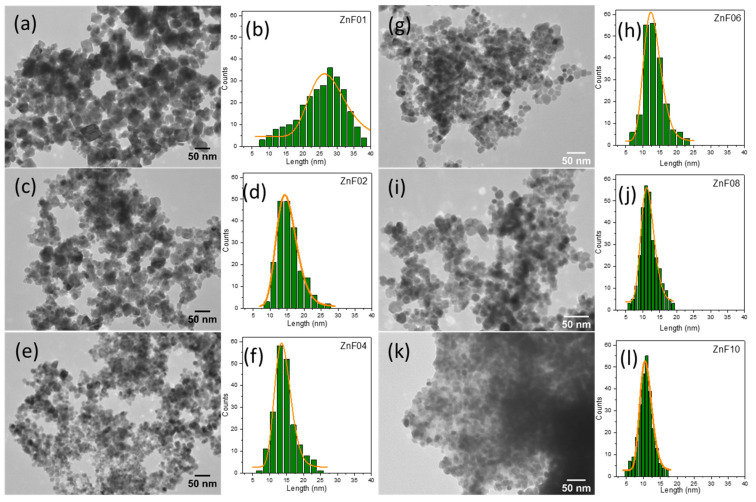
TEM images of ZnF particles and their size distribution histograms fitted to a log-normal distribution (orange lines): (**a**) and (**b**) ZnF01, (**c**) and (**d**) ZnF02, (**e**) and (**f**) ZnF04, (**g**) and (**h**) ZnF06, (**i**) and (**j**) ZnF08, (**k**) and (**l**) ZnF10.

A series of Zn substituted Fe_3_O_4_ particles were fabricated, employing the polyol synthesis route at high temperature and high pressure. The ZnF particles were imaged by TEM, while their size distribution histograms were determined from 200 particles per sample on TEM images (using ImageJ software) and fitted by a log-normal function (using Origin software), resulting in the distribution curves shown in Figure 1. The zinc doping level inside the Fe_3_O_4_ structure was controlled by using different ratios of zinc to iron in the starting precursors. Thus, six different samples were prepared, starting with a Zn/Fe molar ratio of 0.1, 0.2, 0.4, 0.6, 0.8, and 1.0, the resulting Zn-doped Fe_3_O_4_ MNPs being denoted as ZnF01, ZnF02, ZnF04, ZnF06, ZnF08, and ZnF10, respectively. Variation in the morphology and size of as-synthesized ZnF particles could be observed from TEM images, as shown in Figure 1a–f. It has to be mentioned that without any Zn dopant, the polyol synthesis method enabled the formation of polyhedral Fe_3_O_4_ MNPs, as shown by our group in a previous paper [47]. The well-faceted polyhedral shape of particles was preserved for most of the particles when a small amount of Zn precursor was added to the reaction mixture (Figure 1a). The mean average length of polyhedral ZnF01 particles was around 27 nm (Figure 1b and Table 1), much lower than that of polyhedral Fe_3_O_4_ MNPs which was estimated around 46 nm [47]. The size distribution histogram of ZnF01 particles was broad (Figure 1b) and suggests the presence of a second population of particles with a smaller mean size around 15–16 nm. With increasing the Zn precursor to 0.2 mmol, most of the ZnF02 particles lost their polyhedral shape, becoming irregular, while their size distribution was narrower with a mean average size of around 15 nm (Figure 1c,d, Table 1). As greater amounts of Zn precursor were used in the reaction mixture, the resulting particles exhibited irregular and sub-rounded shapes, with a mean average length around 14 nm and narrow size distribution (Figure 1d,e; Table 1). The ZnF particles started to adopt a spherical shape when the Zn precursor concentrations were increased to 0.6 mmol (Figure 1g). The size distribution was kept narrow, while the mean average length slightly decreased around 13 nm concerning the ZnF02 and ZnF04 particles (Figure 1h, Table 1). For the Zn/Fe molar ratio of 0.8 and 1.0, the TEM images revealed that most of the particles had an overall spherical shape, with some being sub-rounded (Figure 1i,k). ZnF08 and ZnF10 particles displayed a much-narrowed size distribution, the mean average length decreasing to 11.5 and 10.8 nm, respectively (Figure 1j,l, Table 1).

As presented above, a very small amount of Zn in the reaction mixture (Zn/Fe = 0.1) led to a drop of the mean length by almost a half, while preserving the shape. The decrease of the mean average length continued at a Zn/Fe molar ratio of 0.2. Starting with a Zn/Fe molar ratio of 0.4, the size of the ZnF particle suffered a progressive decrease by 1 nm at each batch, exhibiting a difference of 5 nm between ZnF04 and ZnF10 particles size. Besides the small variation of size and narrowing the size distribution, the ZnF particles shape was affected, going from a polyhedral to a spherical one. A literature review reveals that the thermal decomposition technique enables the formation of Zn substituted MNPs, exhibiting a well-defined shape that is not affected when modifying the Zn dopant concentration within their structure [31,32]. Instead, the rest of the synthesis methods employed for the elaboration of Zn ferrites, as co-precipitation, microwave-assisted, hydrothermal, and polyol [34,36,37,38,39,42,43,44,45], have shown important morphological modifications of the resulting ZnF particles. A decrease in their size and variations in their shapes were observed when the Zn dopant level within the spinel structure was increased. It has been shown, however, that the systematic decrease of ZnF particle size with increasing the Zn content is a result of obstructing the crystal growth in spinel structure [49].

### 3.2. Structural Properties

**Figure 2:**
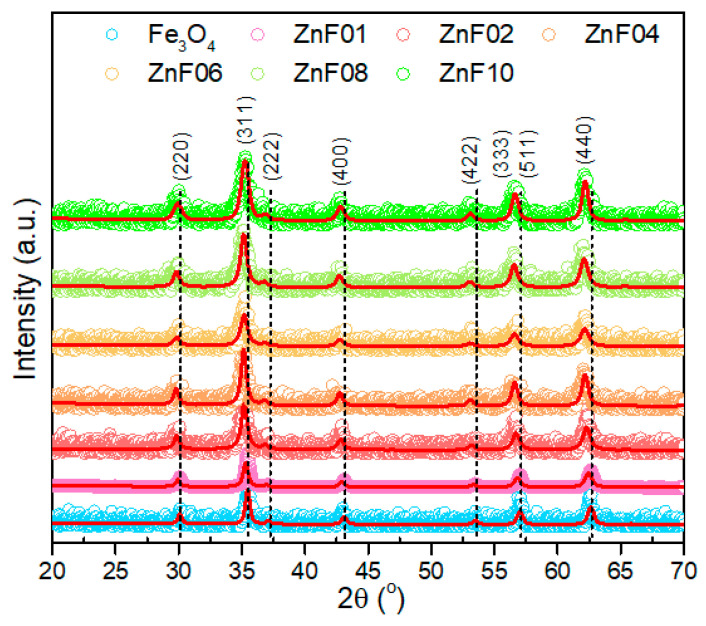
XRD diffraction patterns of Fe_3_O_4_ and ZnF particles. The dashed black lines indicate the progressive shift of all diffraction peaks towards lower angles. The diffractograms were shifted for clarity.

The crystalline structure of ZnF particles was evaluated by X-ray diffraction (XRD) performed on powder samples. As a reference, the diffraction pattern of pure Fe_3_O_4_ MNPs, without any Zn dopant, was included. The XRD patterns corresponding to the ZnF particles are identical to that of pure Fe_3_O_4_ MNPs, confirming the presence of magnetite in all diffractograms (Figure 2). The peaks assigned to (220), (311), (222), (400), (422), (333), (511), and (440) crystallographic planes, which reveal the existence of a single-phase cubic spinel crystalline structure, are well defined, being the only ones present in the diffractograms. For all six samples, no other peaks, for instance, corresponding to zincite (ZnO), were detected within the limit of observation in their XRD patterns, suggesting that the Zn is incorporated in the crystalline structure of magnetite. The vertical black dashed lines in Figure 2 clearly reveal that the diffraction peaks were progressively shifted towards lower angles when going from ZnF01 to the ZnF10 sample. This effect was also observed in other studies, and it was attributed to the positioning of Zn^2+^ ions in tetrahedral sites of the spinel crystalline structure upon synthesis [34,36,40,42,46]. The insertion of Zn^2+^ ions, which have a larger radius than trivalent Fe^3+^, in tetrahedral sites, expands the crystalline structure of ZnF particles, leading to an increase in the lattice parameter. Indeed, according to Rietveld refinements of XRD patterns, the obtained lattice constants gradually increased from ZnF01 to ZnF10 particles (Table 1). This suggests that the increase of the Zn/Fe molar ratio in the reaction mixture increased the Zn dopant content in the structure of the particles. In addition, the diffraction peaks gradually broaden when going from ZnF01 to ZnF10 sample (Figure 2), which implies a decrease of the average crystalline size (Table 1) as calculated using Scherrer’s formula by Gaussian fit of the peaks (220), (311), and (440). As depicted in Table 1, for ZnF01 particles, the average diameter (D_TEM_) obtained from TEM histograms is higher than that given by Scherrer’s formula (D_XRD_), suggesting that the majority of the particles are polycrystalline. Instead, the particles from ZnF02 to ZnF10 samples, with relatively narrow size distribution histograms, are single crystals, as their D_TEM_ are close to the corresponding D_XRD_ (Table 1).

### 3.3. FT-IR Spectroscopy

**Figure 3:**
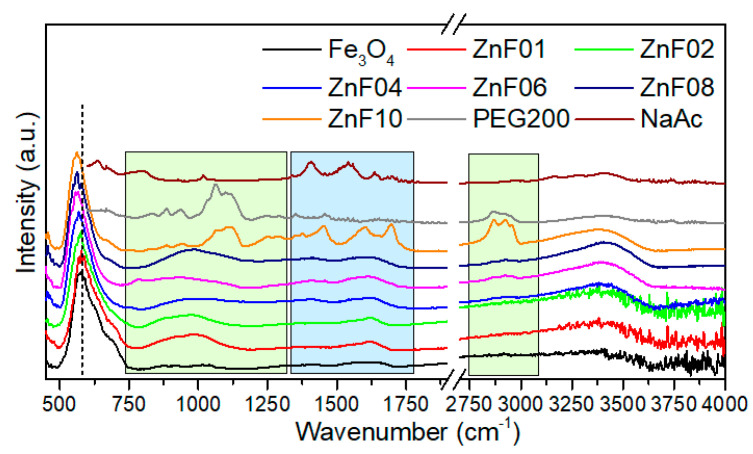
FT-IR spectra of Fe_3_O_4_, ZnF particles, PEG200, and sodium acetate. The spectra are normalized to the highest absorption band and shifted for clarity. The dashed line indicates the shift of the stretching mode of the Fe-O bond. The green and blue rectangles delimit the area of vibrational bands of PEG and acetate, respectively.

When investigating transitional metal-doped magnetite nanoparticles, the FT-IR analysis is very useful as it offers important information on the metal-oxygen stretching modes of spinel ferrites. On the other hand, FT-IR spectroscopy identifies the presence of organic molecules at the surface of the ZnF particles. Figure 3 shows the FT-IR spectra of the ZnF particle series, along with those of PEG200 and NaAc. FT-IR spectrum of Fe_3_O_4_ MNPs, without any Zn dopant, was also included as a reference. This spectrum is dominated by the vibration mode located at 576 cm^−1^, which defines the stretching mode of the Fe^3+^-O bond in tetrahedral sites and confirms the formation of spinel structure. As indicated by the dashed line in Figure 3, the dominant vibration mode shifts towards lower wavenumbers with increasing Zn concentration, from 574 cm^−1^ for the ZnF01 sample to 560 cm^−1^ for ZnF10 particles. This trend was interpreted in the literature in the light of a gradual substitution of Fe^3+^ ions by Zn^2+^ ones in tetrahedral sites of the spinel structure [36,39]. The light green and blue rectangles in Figure 3 delimit the regions of the FT-IR spectrum dominated by absorption bands of PEG200 (750–1250 cm^−1^, 2750–3100 cm^−1^) and acetate (1350–1750 cm^−1^), respectively. These absorption bands are feebly present in the FT-IR spectrum of Fe_3_O_4_ MNPs, suggesting that very small amounts of PEG200 and acetate are present on their surface. In the case of the ZnF01 sample, these absorption bands were visible as broadbands, indicating the presence of a thin coating layer of PEG200/acetate around them. Similar absorption bands were observed for the ZnF02, ZnF04, ZnF06, and ZnF08 samples. Instead, the FT-IR spectrum of the ZnF10 sample exhibited the most relevant absorption bands of both PEG and acetate (Figure 3). As depicted in the TEM images (Figure 1k and Figure S1), the ZnF10 particles were embedded in a PEG200/acetate matrix, and therefore the organic moieties were easily detected by FT-IR spectroscopy. On the contrary, the large-scale TEM images of the rest ZnF particles (Figure S1) showed a thin layer of PEG200/acetate around them, which was faintly detected by FT-IR spectroscopy.

### 3.4. Magnetic Properties

**Figure 4:**
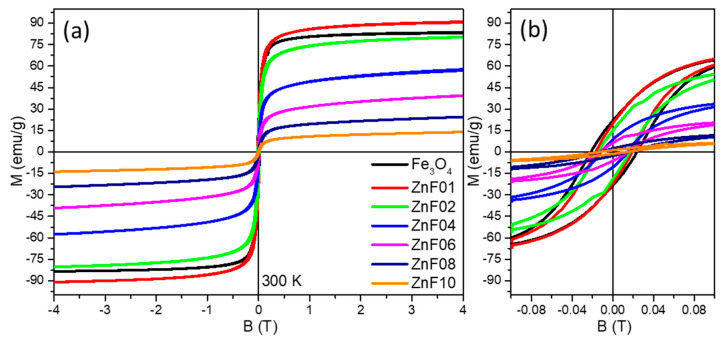
(**a**) Hysteresis loops of Fe_3_O_4_ and ZnF particles at 300 K; (**b**) Low-field regime hysteresis loops.

Hysteresis (M-H) loops of all six batches of ZnF particles were measured at 300 K (Figure 4a), and the values of the main magnetic parameters as the saturation magnetization reachable (M_s_) by the MNPs in the presence of an applied magnetic field, the coercive field (H_c_), and the magnetic remanence (M_r_) were estimated and collected in Table 2. As depicted in the low-field region (Figure 4b), a hysteresis loop exists for ZnF01 particles, indicating ferrimagnetic behavior at RT with remanence and coercivity. The H_c_ value of ZnF01 particles is lower than that of pure Fe_3_O_4_ MNPs without any Zn dopant (Table 2) [49]. Despite the smaller mean average length of ZnF01 particles, they exhibited a higher M_s_ (90 emu/g) value as compared to pure Fe_3_O_4_ MNPs (83 emu/g). Based on the above empirical observations, it might be assumed that the increase in M_s_ is a consequence of the incorporation of the divalent Zn^2+^ ions at the tetrahedral sites of the spinel structure of Fe_3_O_4_ particles. In the case of ZnF02 particles, the H_c_ value decreased to 16 mT while their M_s_ of 80 emu/g is closed to the value of pure Fe_3_O_4_ MNPs (Figure 4 and Table 2). Since the size of ZnF02 particles, with an irregular shape, is almost three times lower than those of pure polyhedral Fe_3_O_4_ MNPs, their high M_s_ value can be attributed as well as to the preference of divalent Zn^2+^ ions towards tetrahedral sites. The next two samples, ZnF04 and ZnF06, exhibited a H_c_ value of 14 and 13 mT, respectively (Table 2), denoting a ferrimagnetic character at RT. The last two samples, ZnF08 and ZnF10, displayed low values of H_c_ and M_r_ (Table 2), which suggest that they are in a superparamagnetic (SP) state at RT. The progressive reduction of H_c_ value could be due to a decrease of the magnetocrystalline anisotropy of ZnF particles, as their shape migrated from polyhedral to spherical one with increasing the Zn/Fe ratio in the synthesis mixture. In contrast, the M_s_ values of these samples dropped considerably with respect to that of ZnF02 particles (Figure 4 and Table 2). The M_s_ of ZnF04 particles decreased by 30% to 56 emu/g, while the M_s_ of ZnF06 particles was reduced by half to 38 emu/g. The SP particles, from ZnF08 and ZnF10 samples, experienced a much higher reduction of M_s_ to 23 and 13 emu/g, respectively. The small reduction of ZnF particles size and the increase of spin canted effect with increasing the sphericity when going from ZnF02 to ZnF10 samples can contribute to the decrease of M_s_. However, the two causes are not the only ones responsible for the huge alteration of M_s_. It is quite obvious that starting with ZnF04 samples, the increase of Zn concentration led to a decrease of the net magnetization, as explained by other groups based on Yafet and Kittel three sub-lattices model [50].

From the six batches of ZnF particles, the first two batches (ZnF01 and ZnF02) exhibit the highest M_s_, which has been attributed to the incorporation of Zn^2+^ ions at the tetrahedral sites of the spinel structure. Since Zn^2+^ ions are diamagnetic, they do not contribute magnetically to the enhancement of the particles’ magnetic moment. The major role of Zn^2+^ ions is given by their special preference to occupy the tetrahedral sites of the spinel structure, increasing thus the number of Fe^3+^ ions at the octahedral sites. According to the literature, this implies a certain Zn doping level, x, in the general formula of Zn_x_Fe_3−x_O_4,_ in between 0.2 and 0.4 [26,27,28,29,42,43,44]. It has also been shown that the initial Fe/Zn molar ratio of precursors used in the synthesis is not preserved in the ZnF particles—usually, the incorporated Zn^2+^ ions amount is less [51]. In these conditions, we have adopted a rough method to quantify the amount of incorporated Zn. As presented in Section S2, our method is based on the determination of iron concentration from the six samples having identical mass and on the assumption that the PEG layer around the ZnF particles represents 10–15% of the total sample mass. We have found that the Zn doping level increased from 0.21 (±0.6) for ZnF01 particles to 1.23 (±0.4) for ZnF10 (Figure S2b, Table S1). Thus, a difference between theoretical x and the experimental estimated one is recorded (Figure S2b, Table S1). This difference is small for the first two samples but became larger with increasing the amount of Zn precursor in the reaction mixture. The x values of 0.21 (±0.6) and 0.39 (±0.6) found for ZnF01 and ZnF02 samples, respectively, explain their high recorded values of M_s_.

The ZnF particles from ZnF01 to ZnF06 samples exhibit an important value of coercivity at RT, which suggests that they are prone to form clusters in colloidal solutions as demonstrated in the case of polyhedral Fe_3_O_4_ MNPs [52]. To reveal the degree of clusterization, we have performed dynamic light scattering determination. The hydrodynamic diameters of ZnF particles from ZnF01 to ZnF06 samples, at a very low concentration of 0.01 mg_MNPs_/mL, were similar and about 395 nm, while the ZnF08 and ZnF10 samples were 245 nm and 240 nm, respectively (Figure S3a and Table S2). These values are much greater than the average size resulting from TEM images (Figure 1) and indicate that the ZnF particles from all batches form clusters in the colloidal suspension. The polydispersity index (PDI) values are between 0.17 and 0.36 (Table S2), which indicates a quasi-narrow size distribution of clusters. By increasing the colloidal concentration ten times to 0.1 mg_MNPs_/mL, the hydrodynamic diameters increased for the majority of ZnF samples, exception made the ZnF06 particles, whose hydrodynamic diameter remained constant. For the first three samples, ZnF01, ZnF02, and ZnF04, the hydrodynamic diameter of clusters increased by 200 nm, while the PDI decreased considerably (Table S2) indicating a narrowing of the clusters size distribution (Figure S3). In the case of ZnF08 and ZnF10 samples, the hydrodynamic diameter increased by 180 nm and 100 nm, respectively (Table S2). The PDI increased as well, indicating a broadening of the clusters size distribution (Figure S3 and Table S2). The magnetic dipolar interactions manifested between ZnF particles from ZnF01 to ZnF06 samples are the main driving force that facilitates clusterization. Due to their coercivity, the ZnF particles cannot be individually dispersed within the colloidal solutions. These ZF particles self-aggregate in clusters that comprise several tens of particles, which by increasing the colloidal concentration extend their size. Instead, in the case of ZnF08 and ZnF10 samples, which are close to a superparamagnetic state, it seems that the clusters are developed during synthesis being facilitated by the PEG coating. TEM analysis (Figure 1 and Figure S1) revealed ZnF particles interconnected throughout the PEG layer. Overall, the ZnF particles from all six batches, independently on their magnetic states, prefer to stabilize colloidally in clusters, which might impact their hyperthermia performances.

### 3.5. Hyperthermia Properties

In the following, the magnetically induced heating capabilities of ZnF particles from all batches were investigated in water at two different concentrations: 4 mg_Fe_/mL and 1 mg_Fe_/mL. The heating curves, shown in Figure S4, were fitted with the Box–Lucas function and the resulting parameters were used to evaluate the specific absorption rate (SAR), following the procedure detailed in Section S5. The SAR values were expressed in watts per unit mass of iron (W/g_Fe_), the iron concentration of ZnF particles in aqueous solutions being determined with thiocyanate assay and used for data normalization. The SAR values were plotted as a function of the amplitude (H) of the applied AMF ranging from 5 kA/m to 65 kA/m at a fixed frequency of 355 kHz. The contribution from pure water at each H was measured and subtracted as the background.

**Figure 5:**
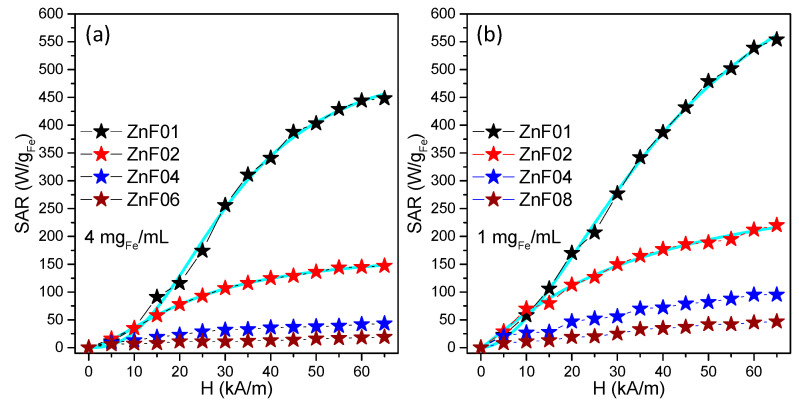
Specific absorption rate (SAR) dependence on the AMF amplitude (H) for ZnF particles dispersed in water at two different concentrations: (**a**) 4 mg_Fe_/mL and (**b**) 1 mg_Fe_/mL.

Figure 5 summarizes the obtained mean SAR values for ZnF particles samples presented in this study. According to magnetic characterization, the ZnF01 particles are, at RT, in a ferromagnetic state with a H_c_ of 18 mT (14.3 kA/m). Therefore, the typical evolution of SAR values with H presents a sigmoidal shape that can be fitted phenomenologically with a simple logistic function (Section S7, Table S3), as shown in our previous papers [23,45,47]. In other words, the main contribution to SAR is given by the energy losses quantified by the area of the dynamic hysteresis loops. For H values of 5 and 10 kA/m, the SAR values are very low, as the hysteresis areas are insignificant. The hysteresis area is constantly increasing for H values in between 15 and 45 kA/m, resulting in a linear increase of SAR values up 400 W/g_Fe_. By further increasing the H (50–65 kA/m), the SAR tends to saturate around 450 W/g_Fe_, as the hysteresis area does not increase significantly. When the iron concentration in the sample is decreased four times to 1 mg_Fe_/mL, higher SAR values are recorded (Figure 5b). For the H range between 15 and 45 kA/m, the SAR vs. H slope is steeper, while a considerable increase in SAR is observed starting with H of 45 kA/m, reaching maximum values around 550 W/g_Fe_.

The SAR evolution with H, for ZnF02 particles, follows a similar sigmoidal trend, however, the values are much lower than those given by ZnF01 particles. The SAR values saturate around 140 W/g_Fe_ and 220 W/g_Fe_ at a concentration of 4 mg_Fe_/mL and 1mg_Fe_/mL, respectively. Since there is not a big difference in the magnetic parameters of both samples (Table 2), the high drop of SAR values might be associated with the lower size of ZnF02 particles, whose Brownian contribution is thus reduced as compared to bigger ZnF01 particles. The SAR values of the next two samples, ZnF04, and ZnF06 particles are very poor as compared to the previous two samples. Over the entire range of H, the SAR values slightly increase from 10 to 40 W/_Fe_ and 5 to 20 W/g_Fe_ for ZnF04 and ZnF06 particles, respectively. By decreasing the iron concentration to 1 mg_Fe_/mL, the SAR values double in each case. The heating abilities for the last two samples, ZnF08 and ZnF10, were insignificant and were not taken into consideration.

Among the six samples, the first two, ZnF01 and ZnF02, exhibited the best MH performance. In this regard, to illustrate their potential in MH applications, two types of ZnF particles have been further tested in vitro on cancer and normal cell lines.

### 3.6. Cytotoxicity Studies

The cytocompatibility of the two most promising nanoparticles (ZnF01 and ZnF02) was evaluated in cancerous (A549—human pulmonary cancer cells) and normal cells (BJ—human foreskin fibroblasts) phenotypes by AB and NR assays. Due to the inherited optical and biochemical interferences of the nanomaterials with the viability assays, as a first step, the interferences were evaluated. ZnF01 and ZnF02 particles did not biochemically interfere with the assays, however, an optical interference that could be resolved by a centrifugation step and the measurement of the fluorescent signal on the supernatant was noticed. Similar to these observations, we and other groups previously reported the optical interferences of nanomaterials [47,53,54].

**Figure 6:**
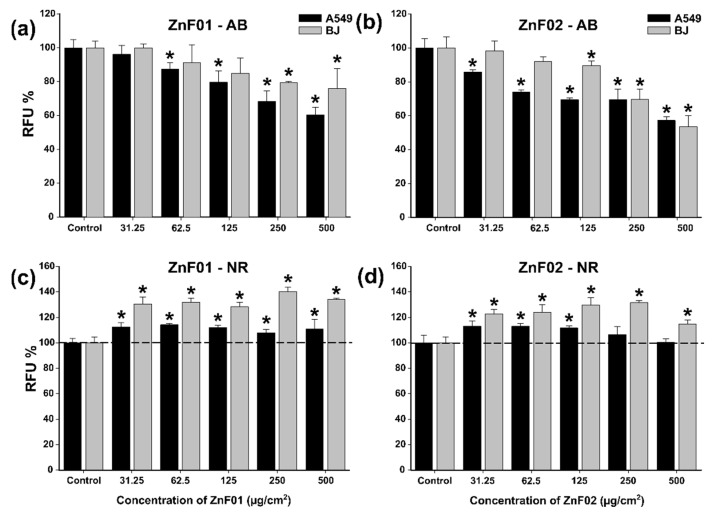
Cytocompatibility of ZnF01 (**b**,**d**) and ZnF02 (**a**,**c**) on A549 and BJ cell lines was evaluated after a 24 h exposure. Cellular viability was evaluated using Alamar Blue (**a**,**b**) and Neutral Red (**c**,**d**) assays. Data were expressed as relative values to the negative control (100%), as mean ± SD of three biological replicates. Asterisks (*) indicate a significant difference compared to the negative control (ANOVA + Dunn’s; *p* < 0.05).

Regarding the cytocompatibility, based on the AB data, both types of nanoparticles displayed moderate toxicity, inducing an approximately 40% reduction of cellular viability at the highest tested dose of 500 µg/cm^2^ (Figure 6a,b). Higher cytotoxicity was observed towards the cancerous cell type ZnF01, displaying a statistically significant effect starting from the dose of 62.5 µg/cm^2^, while for ZnF02, the effect was observed from the dose of 31.25 µg/cm^2^. Conversely, for the BJ cell line, a statistically significant effect was observed only from the dose of 250 µg/cm^2^ for ZnF01 and 125 µg/cm^2^ for ZnF02. On the other hand, NR data indicated a slight increase in viability in the case of both cellular types. This increase was more pronounced for the BJ cell line, viability reaching values between 130 and 140% for ZnF01 and 115-130% for ZnF02 (Figure 6c,d). A small difference between the two types of nanoparticles was observed at the higher doses, as in the case of ZnF01 where a steady plateau was reached while for ZnF02 a decrease in viability was observed for both cell types at the highest tested dose. Based on previous observations, on cancerous and normal cell types, the cellular exposure to nanomaterials induces the formation of autophagosomes that favors the ATP-dependent incorporation of the NR dye in the intracellular compartment and thus increase the observed cellular viability [47,53]. Moreover, these differences in viability could be due to the redox-active surface of MNPs that interferes with the electron flow and mitochondrial functionality and thus decrease the mitochondrial diaphorase-dependent conversion of resazurin to the fluorescent resorufin [55]. Dissimilarities between the results obtained with different assays were previously reported, complementary assays being recommended for the evaluation of the toxicity of the nanoparticles [56,57]. Similar to the present results, Könczöl et al. reported that exposure to Fe_3_O_4_ MNPs resulted in an increase in viability according to the NR assay and a slight decrease in viability according to the WST assay, the latter one being an assay similar to the AB assay [54].

Three-way ANOVA with the variables dose, type of nanomaterial, and type of cell indicates that all factors influence the measured viability by the AB and NR assays. The AB data revealed significant differences between the cell types at low doses (31.25, 62.5, and 125 µg/cm^2^), while at the higher doses no statistical significance was observed. The type of nanoparticle did not induce significant differences in data, with both particles displaying similar cytotoxic profiles. Based on the NR data, the three-way ANOVA analysis revealed significant differences in viability between the two cell types for both ZnF01 and ZnF02 particles at the highest tested doses of 250 and 500 µg/cm^2^.

The present ZnF particles displayed a lower cytotoxic potential than our previously synthesized ZnF MNPs by the polyol-mediated process. In the previous study, upon incubation with 200 µg/mL (equivalent to 125 µg/cm^2^), the viability of three different types of cancerous cells decreased to approximately 50%, while in the case of normal cells the recorded viability was 80% [45]. Compared to ZnF01 and ZnF02 particles, the previously reported Zn doped Fe_3_O_4_ had a higher Zn content being coated with ethylene glycol. Moreover, the higher toxicity observed could have been the result of the formation of the ZnO phase that is known to be highly toxic [58]. Even though Zn is an oligo element needed in the cellular processes, high concentrations can induce cytotoxicity. Moise et al. evaluated the biocompatibility and the cellular magnetic response of biogenic Zn-doped Fe_3_O_4_ nanoparticles and reported that cytotoxicity increases in line with the doping level [59].

Congruent with the current results, Wang et al. obtained similar cytotoxicity for synthesized ZnF MNPs coated with silica and a diameter of 22 nm [33]. The authors evaluated the toxicity in two different cell types, at concentrations ranging from 50 to 1000 µg/mL (equivalent to 12.5 to 250 µg/cm^2^), and observed a statistical decrease of viability starting from an exposure dose of 150 µg/cm^2^ [33]. For uncoated spherical ZnF particles with an average size of 40 nm, a strong decrease in the cellular viability of cancerous cells was observed. At an exposure dose of 40 µg/mL (equivalent to 25 µg/cm^2^), the authors reported viabilities ranging from 25 to 40% [60]. Conversely, Yang et al. reported that ZnF particles with an average diameter of 26.5 nm coated with phosphorylated PEG display high biocompatibility, reducing the cellular viability of MCF-7 cells by less than 20% at a dose of 200 µg/mL. Moreover, less cytotoxicity was observed for these types of nanoparticles than for phosphorylated PEG Fe_3_O_4_ nanoparticles [61]. Similar to these observations, silica-coated Zn-MNPs with a diameter of 22 nm and polysaccharide-coated Zn-MNPs with a diameter of 10 nm showed a good cytocompatibility at doses of up to 250 µg/cm^2^ and 170 µg/cm^2^, respectively [62,63].

Even though optimizing the magnetic properties of MNPs by increasing the doping level is a viable approach for obtaining MNPs with higher potential in MH treatment, the cytocompatibility should be maintained. In this regard, several studies revealed that the surface coating can increase the cytocompatibility of MNPs and make them more suitable for cellular applications [64,65].

### 3.7. Evaluation of Cellular Uptake

**Figure 7:**
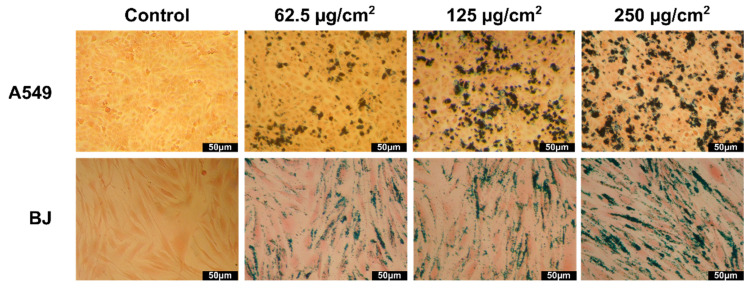
Microscopic images of A549 (**upper** panels) and BJ (**lower** panels) cells incubated with 62.5, 125, and 250 μg/cm^2^ ZnF01. Nanoparticles were stained with Prussian Blue.

**Figure 8:**
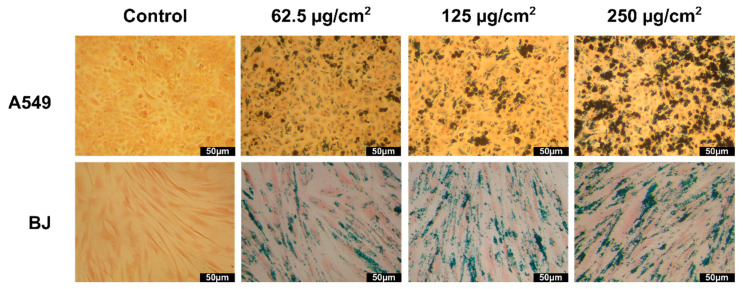
Microscopic images of A549 (**upper** panels) and BJ (**lower** panels) cells incubated with 62.5, 125, and 250 μg/cm^2^ ZnF02. Nanoparticles were stained with Prussian Blue.

The ability of the ZnF01 and ZnF02 particles to be internalized into the cellular compartment, and thus to act as heat mediators in MH applications, was firstly evaluated qualitatively by the Prussian Blue stain. Microscopic images of both cell types (A549 and BJ) after an incubation of 24 h with the ZnF particles were taken (Figure 7 and Figure 8). Both types of ZnF particles were highly internalized in a dose-dependent manner, while the cellular morphology was not affected by the exposure. Independent on the dose testing, cells preserved their morphology with no decrease of the cellular volume, indicative of a cytotoxic effect being observed. Regarding the uniformity of the internalization, as in other similar studies, the nanoparticles were not taken up uniformly, some cells displaying a cytoplasmatic overload, while others had a lower number of internalized nanoparticles [66,67,68]. This phenomenon appeared to be more pronounced in the case of ZnF01 particles, most probably due to their higher H_c_ and M_s_ that favor the agglomeration and formation of clusters. Both types of cells internalized high quantities of ZnF particles in their cytoplasmic volume, with no evident difference in internalization being observed on the microscopic images. Higher magnification images revealed that the ZnF particles were distributed within the cytoplasm, but not in the nucleus.

**Figure 9:**
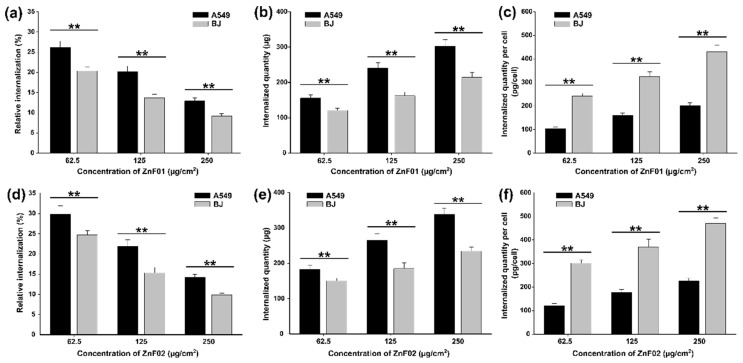
Cellular internalization of ZnF01 (**a**–**c**) and ZnF02 (**d**–**f**) in A549 and BJ cells after a 24 h exposure; (**a**,**d**) the relative internalization; (**b**,**e**) the total amount of ZnF01/ZnF02 internalized in A549 and BJ cells; (**c**,**f**) the internalized quantity of ZnF01/ZnF02 per cell. The values are expressed as mean ± SD of at least three biological replicates. Double asterisks (**) indicate significant differences (ANOVA + Dunn’s; *p* < 0.001).

For the quantitative evaluation of the internalization, the reaction of digestion-free ferric ions with thiocyanate was performed on cells exposed to doses of 62.5, 125, and 250 μg/cm^2^. Independent on the cell type, the highest relative internalization was observed at the lowest dose. From the total exposure dose of 600 µg (62.5 µg/cm^2^, 9.5 cm^2^), approximately 25% and 30% of ZnF01 and ZnF02 particles, respectively, were internalized in A549 while 20% and 25% in BJ. As the dose increased, the relative internalization decreased in a dose-dependent manner (Figure 9a,d). These results are in agreement with those published by our group on polyhedral—Fe_3_O_4_ MNPs, for which the relative internalization decreased as the dose increased from 50 to 1000 µg/mL [47]. Moreover, the relative internalization in A549 cells and normal gingival fibroblasts mirror the present results, as the values were between 15 and 30% [47].

Although the relative internalization was inversely proportional to the exposure dose, the total internalized quantity (Figure 9b,c) and the quantity per cell (Figure 9e,f) increased as a function of the dose. The total internalized quantities of ZnF01 particles per well in A549 cells increased from 150 µg to 300 µg and from 120 µg to 215 µg for BJ cells. For ZnF02 particles, the internalization increased from 180 µg to 340 µg in the case of A549 and from 150 µg to 235 µg for BJ cells. Statistically significant differences were observed in the internalization between the two cell types, the A549 cells displaying a higher capacity to internalize both ZnF01 and ZnF02 particles. These results are in agreement with previously published data from our group, where an increased uptake of nanoparticles was observed for the A549 cells in comparison with normal fibroblasts [47]. As cancerous cells display an increased demand for nutrients, they generally exhibit increased endocytic activity and nanoparticle uptake, this preferential internalization being reported in most of the studies from the literature [5,69]. This higher total internalization is an important parameter in the current study, as in other MH studies, because this quantity influences the heat generation after applying the AMF.

A three-way ANOVA with the type of nanoparticle, type of cell, and the dose as factors revealed that all factors contribute in a significant manner to the observed total internalized quantity (*p* < 0.001). Independent on the dose, using a higher quantity of internalized particles was more observed in A549 cells than in BJ cells. Moreover, ZnF01 particles had a significantly lower internalization than ZnF02, independent of the cell type and the dose tested. In comparison with ZnF01, ZnF02 particles have a smaller size that could have favored the internalization. The dependency between cellular uptake and size is a well-known fact in nanotoxicology and has been previously reported [70,71,72]. Using MCF-7 and HeLa cells, Guo et al. reported that the cellular internalization of MNPs was dependent on their size, MNPs with a diameter of 60 nm achieving a 1.7-, 1.9-, and 5.2-folds higher internalization than MNPs with a diameter of 120, 200, and 310 nm, respectively [73]. Similarly, Shapero et al. reported that the internalization of silica nanoparticles is dependent on their size, nanoparticles with lower size having a higher internalization rate [74]. In addition, the more sub-rounded shape of the ZnF02 particles could have increased the cellular internalization, as spherical nanomaterials display, in general, a higher internalization. In comparison with nanorods, nanospheres of amino-surface modified MNPs showed better internalization efficiency in L929 cells [75]. Similarly, gold nanospheres achieved a higher cellular internalization than gold nanorods [71].

For a better comparison of the present data with data from the literature, the cellular uptake was also expressed as the ratio between the total quantity of ZnF01 and ZnF02 internalized per well and the cellular number. When normalized on the cell number, higher quantities of nanoparticles were observed in BJ cells. Due to their higher surface and cytoplasmic volume, BJ cells internalized on average 2–2.5 times more ZnF01 and ZnF02 nanoparticles than their cancerous counterpart (Figure 9c,f). In a recent study, Wang et al. evaluated the cellular uptake and hyperthermia performance of Zn-MNPs coated with silica and reported that the cellular internalization increases as the exposure duration increases [33]. After a 12 h incubation with an exposure dose of 200 µg/mL (equivalent to 50 µg/cm^2^), osteosarcoma (MG-63) cells internalized a Fe^3+^ quantity of 60 pg/cell [33]. The reported value is close to the current results for cancerous cells, at an exposure dose of 62.5 µg/cm^2^ and exposure for 24 h, the Fe^3+^ content per A549 cell being approximately 60 pg/cell and 65 pg/cell for ZnF01 and ZnF02 particles, respectively.

### 3.8. In Vitro Magnetic Hyperthermia

Before the performance evaluation of the MH treatment in inducing cellular death in cancerous cells, the influence of the AMF alone was evaluated on both cell types at a frequency of 355 Hz and the intensities of 30, 45, and 60 kA/m. Following our previously published results and other results from the literature, this exposure was accompanied by a modest increase in temperature, not exceeding 0.5 °C. This temperature rise did not affect cellular viability [47,76,77]. However, when cells pre-incubated with ZnF01 and ZnF02 were exposed to the AMF, important temperature increases were noticed (Figures S6 and S7).

**Figure 10:**
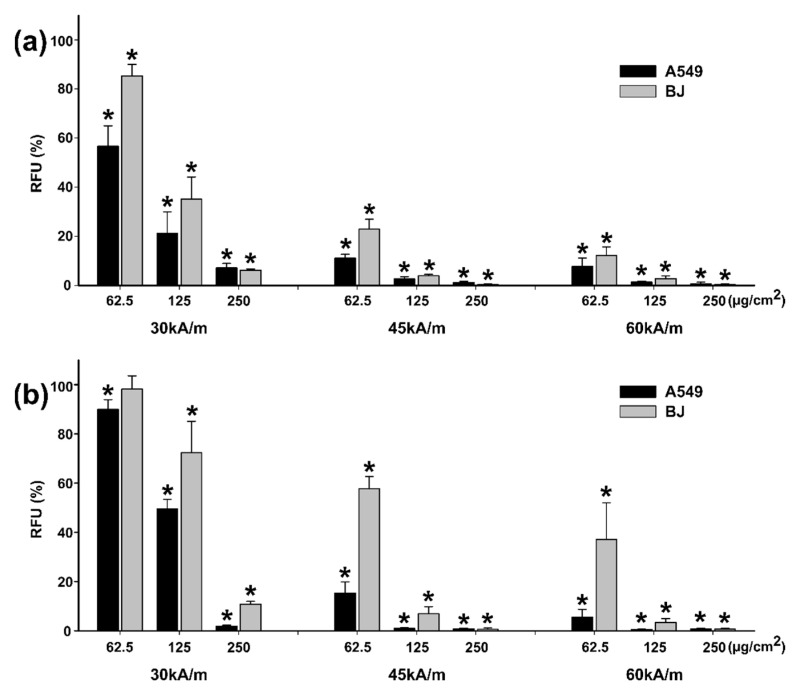
Cytotoxic effects of internalized ZnF01 on A549 and BJ cell lines were evaluated after a 30 min exposure to three different values of H (30, 45, 60 kA/m). Cellular viability was evaluated using Alamar Blue (**a**) and Neutral Red (**b**) assays. Values were expressed as relative values to AMF negative control (100%), as mean ± SD of three biological replicates. Asterisks (*) indicate a significant difference compared to the negative control (ANOVA + Dunn’s; *p* < 0.05).

**Figure 11:**
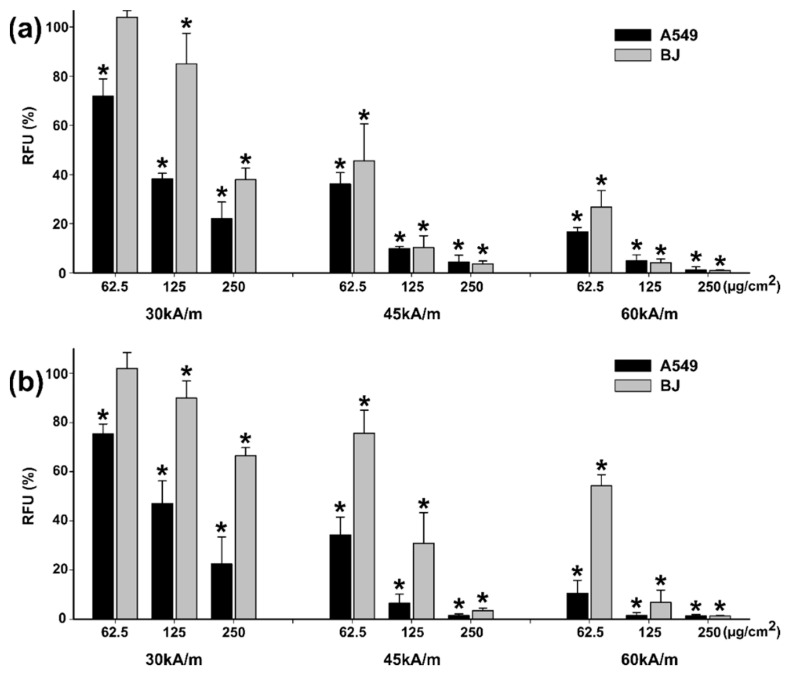
Cytotoxic effects of internalized ZnF02 on A549 and BJ cell lines were evaluated after a 30 min exposure to three different values of H (30, 45, 60 kA/m). Cellular viability was evaluated using Alamar Blue (**a**) and Neutral Red (**b**) assays. Values were expressed as relative values to AMF negative control (100%), as mean ± SD of three biological replicates. Asterisks (*) indicate a significant difference compared to the negative control (ANOVA + Dunn’s; *p* < 0.05).

Similar to the observations made in the cytotoxicity studies, the AB assay yielded results that indicate higher cytotoxicity when compared to the NR assay results (Figure 10 and Figure 11). Starting from the lowest dose of 62.5 µg/cm^2^ ZnF01 particles and the lowest intensity field-tested of 30 kA/m, a decrease in viability was observed for both cell types in the case of AB assay (Figure 10a).

The viability decreased to approximately 55% in the case of A549 cells, while for the BJ the recorded viability was 85%. The viabilities decreased by increasing the exposure dose, as the internalized quantity of ZnF01 particles increased. At the highest tested dose, the viability was below 10% for both cell types. By increasing the intensity of the AMF, the effects were more pronounced, and the viability was below 15% at all concentrations tested at the maximum intensity of the AMF (Figure 10a). The same observations were made with the NR assay, the efficiency of the MH treatment increasing in a dose- and field intensity-manner. However, based on this assay, the cytotoxic effects were less pronounced on the BJ cells, being in line with the observations made in the cytotoxicity testing. At the dose of 62.5 µg/cm^2^, and for field intensities of 45 and 60 kA/m, the recorded viabilities were 57.8 and 37.1% for BJ, while for A549 they were 15 and 5% (Figure 10b).

Congruent with the results obtained on the ZnF01 particles, MH treatment with ZnF02 particles induced cellular death in a dose- and field intensity-manner (Figure 11). At 30 kA/m and an exposure dose of 62.5 µg/cm^2^, a statistically significant decrease in viability was noticed on the A549 cell line, while on the BJ cell line no effect was observed (Figure 11a,b). At the next two exposure doses, a difference in cellular death was observed between the two cell types. For A549 cells, the recorded viabilities using AB assay were 38% and 22%, while for BJ they were 85% and 37%. The results obtained by NR assay mirror the AB results, the viabilities being 47% and 22% for A549 and 89% and 66% for BJ cells (Figure 11b). Based on the AB data, at higher intensity fields, independent of the exposure dose, similar cellular death was observed for the two cell types, excepting the dose of 62.5 µg/cm^2^. As in the case of ZnF01, the results obtained with the NR assay for ZnF02 particles indicate a slightly lower efficiency of the MH treatment (Figure 11b), but higher cytotoxicity towards the cancerous cell phenotype.

For each intensity field, a Three-Way ANOVA analysis with the cell type, nanoparticle type, and dose as factors, and the data sets from the AB and NR assays as the experimental data was performed. Statistical analysis revealed that at all intensity fields, independent of the viability assay used, all three factors influenced the viability values, with statistically higher toxicity being observed in cancerous cells. Moreover, between the particles tested, ZnF01 particles were more efficient in inducing cellular death.

Compared with our previously published study, where the MH treatment efficiency of polyhedral Fe_3_O_4_MNPs was evaluated [47], higher temperatures and higher cellular deaths were observed hereby. In the previous study, cells were exposed for 24 h to Fe_3_O_4_MNPs at a concentration ranging from approximately 10 µg/cm^2^ to 200 µg/cm^2^, subsequently washed to remove non-internalized MNPs and exposed to similar AMFs. While at the lowest field intensity of 30 kA/m a 50% decrease in the A549 cellular viability was observed only at the highest dose (200 µg/cm^2^) of Fe_3_O_4_MNPs, ZnF01 and ZnF02 particles decreased the cellular viability by 90% and 80% at an exposure dose of 250 µg/cm^2^. Similarly, at higher intensity fields, an increased cytotoxic potential was observed for ZnF01 and ZnF02 particles. In comparison with the polyhedral Fe_3_O_4_ MNPs, the current particles displayed a slightly higher internalization and cytotoxicity, explaining these results.

In a recent study, Wang et al. reported the MH treatment (H = 10–14 kA/m, *f* = 430 kHz) of human osteosarcoma (MG-63) cells exposed to 400 µg/mL (100 µg/cm^2^) of silanized Zn-MNPs [33]. When the cells were exposed to the entire dose of nanoparticles, temperatures reaching 43–46 °C induced marked cellular death and, depending on the incubation time, the percentage of viable cells varied from 22% to 50%. As stated by the authors, these impressive results were most probably due to the local/environmental temperature rise and not completely related to the direct interaction of MNPs with cells [33]. Conversely, and with more significance to the current study, when cells were exposed to the AMF only with the internalized quantity of MNPs, the percentage of viable cells was around 90% [33]. In comparison with the current results, the recorded viabilities for the cancerous cell line (A549) at the exposure dose of 125 µg/cm^2^ were approximately 20% and 40% for ZnF01 and ZnF02 particles, respectively. A straight comparison between the obtained results is not feasible, as the frequency and the intensity of AMF are different. Moreover, variables such as cell number, loading of cells with MNPs, time point after MH treatment, influence the obtained results and the efficiency of MH treatment [5]. The ability of Zn-MNPs to work efficiently in MH treatment was also reported by Lachowicz et al. [63]. In their study, biocompatible Zn-MNPs coated with polysaccharides induced cellular death in cancerous cells at low doses. A 70% decrease in viability for a murine neuroblastoma cell line was observed after a pre-incubation of 24 h with 0.18 mg/mL Zn-MNPs (unbound MNPs were washed after the incubation) and exposure for 15 min at an AMF with an intensity of 33.33 kA/m and a frequency of 360 kHz [63]. Biocompatible Zn-MNPs with increased heating capabilities compared to their Fe_3_O_4_MNPs counterparts are promising candidates for MH treatment, allowing usage of significantly smaller doses of the heating agents than the ones currently used.

**Figure 12:**
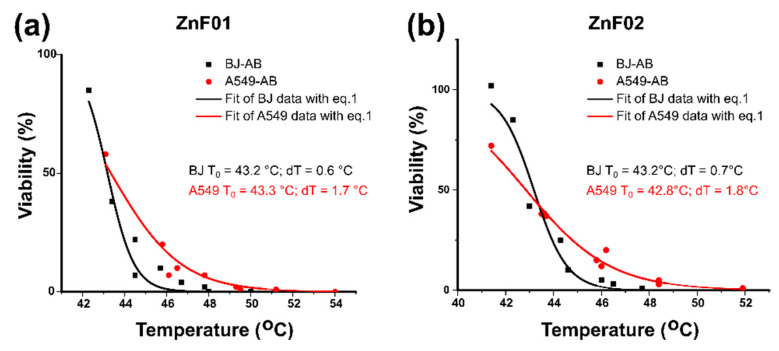
Viability data for BJ and A549 cell lines for ZnF01 (**a**) and ZnF02 (**b**) based on the AB assay, plotted against the saturation temperatures reached during MH and their corresponding fitting curves and fitting parameters based on Equation (1).

For both cell lines, the data obtained using the viability assays could be fitted with a sigmoidal function:

(1) $$C\left(T\right)=\frac{A}{1+e^{\frac{T-T_{0}}{\mathrm{dT}}}}$$

where A represents the viability of control cells (95–100%) and dT quantifies the temperature width for a given decrease in cell viability. T_0_ represents the temperature at which the viability reaches a value of 50%. This equation was derived [78] from a two-state model of temperature-dependent cell damage, as initially proposed by Feng et al. [78]. This simplified form was used for comparing MH heating with endogenous hyperthermia heating, with the condition of having the same time of exposure to high temperatures [79]. As in our case, in all experiments, cells were exposed 30 min to MH, the experimental data were well fitted with the function depicted in equation 1, as can be seen in Figure 12, for both cell types and the two batches of ZnF particles.

The temperature T_0_ represents the saturation temperature, at which half of the cells were killed after they were exposed for 30 min to the MH treatment. In other words, the temperature at which the cells were exposed for 30 min to MH received a 50% lethal dose (LD50%). As one can notice, in all cases the T_0_ values are close to 43 °C. For ZnF02, the T_0_ is slightly higher for BJ cells (43.2 °C) as compared to A459 cells value (42.8 °C), indicating a higher LD50% for BJ cells, thus an apparently higher resistance to the MH treatment for this cell line. For ZnF02 MNPs the T_0_ values are almost identical (within the measurement errors) for the two cell lines. However, the dT parameter behaves identically for the two types of MNPs, being more than double in the case of cancer cells as compared to normal ones. This result means that the decrease in viability occurs in a much larger temperature interval in the case of cancer cells. Even for samples with the same T_0_, a larger dT signifies that the decrease in viability starts at lower temperatures but, on the other hand, reaching 100% cell death requires a higher temperature. In other words, the larger the dT the larger the distribution of cells with different responses to the hyperthermia treatment. Our results suggest that cancer cells have more dispersed sensitivities to the MH treatment, containing both cells more sensitive to the temperature and cells which are more resistant to the same treatment. The fact that most studies report that cancer cells are more sensitive to hyperthermia treatment might be explained by this much larger variability of cancer cells to thermal treatment, and the occurrence of cell subpopulations more sensitive to a temperature rise.

Although the viability data are well fitted by equation 1 showing a clear dependence of viability on the temperature reached during MH, other mechanisms which might be also influenced by the temperature could be involved in the cells’ death. The physical disruption of membrane structure and the increase in the membrane fluidity and permeability were hypothesized to be the mechanisms of action behind the increased susceptibility of cancerous cells to different cytotoxic and cytostatic drugs after combined therapy with MH. On the other hand, the temperature measured by macroscopic probes could differ significantly from the temperature in the near-vicinity of the cellular-internalized MNPs, where higher temperatures are expected. This temperature gradient was previously pinpointed in hyperthermia experiments carried out at similar temperatures, achieved by heating the MNPs loaded cells either under MH or in a water-bath, demonstrated a higher cellular death in the former case [79]. The local increase of the temperature at the surface or in the proximity of MNPs might induce cellular death through lysosomal pathways. As discussed more in detail in a previous paper [47], it has been shown that the potential mechanism in cell death was related to the MNPs accumulation in cell lysosomes and the subsequent lysosome membrane disruption, upon the application of an AMF. Cell damage is an event occurring at the single-cell level, the mechanisms involved being the lysosome membrane permeabilization and ROS formation during AMF treatment. This process is suggested also by our results of the increased NR—a lysosomal dye—accumulation in the cells, when the latter were exposed to MNPs uptake experiments. This preferential lysosome accumulation of MNPs might lead to local heating, and which can eventually trigger the ROS production and the other mechanisms described above, finally leading to cell death. Nevertheless, in a very recent paper [52], TEM analysis on both normal and cancer cell lines revealed a clear MNPs accumulation in lysosomes, thus supporting the lysosomal mechanism of cell death.

## 4. Conclusions

In this study, a series of magnetic nanoparticles with different zinc doping levels were successfully synthesized by the polyol method. Substitution of iron cations with zinc led to increased magnetization saturation values of up to 90 emu/g at low doping values, but as the Zn doping increases both the size and the magnetic performances of the MNPs decrease, emphasizing the need to fine-tune the Zn/Fe ratio for optimal MH results.

Cytocompatibility studies on ZnF01 and ZnF02 (the MNPs with the best magneto-thermal properties) on two cell lines using AB and NR assays revealed moderate or no cytotoxicity, with no significant toxicity difference between the two nanoparticles. The differences in the viability data recorded with the two assays emphasize the need for multiple and complementary assays in the evaluation of nanoparticle cytocompatibility. MNPs were internalized in cells, in a dose-dependent manner, with the smaller ZnF02 displaying a higher cellular uptake. Independent on the nanoparticle type, a significantly increased cellular uptake was present in cancerous cells.

Both types of nanoparticles displayed good heating-inducing capabilities, with intracellular MH experiments revealing high temperatures associated with increased cellular death. Even though ZnF01 displayed a lower cellular uptake than ZnF02, higher temperatures, and higher cellular death were recorded in their case, which can be explained by their higher SAR values. Cancerous cells were less resilient than normal cells to the MH treatment due to the higher MNPs internalization and higher temperatures reached during the MH. The analysis of the viability data during MH as a function of the saturation temperature reached during the MH treatment revealed that, in the case of cancer cells, the viability starts to drop at lower temperatures as compared to normal ones, but at higher temperatures, a subpopulation of cancer cells seems to be more resilient to MH treatment as compared to normal cells. This observation might explain why, in most of the clinical trials, the MH was used in conjunction with radio- or chemo-therapies.

The current results indicate that Zn-substitution represents an efficient strategy to improve the magnetic and heat generation properties of MNPs while preserving biocompatibility. Usage of smaller doses of heating agents than the ones currently used makes these particles promising candidates for MH treatment. Further studies monitoring long-term cytotoxicity and efficiency in vivo are needed to evaluate the clinical potential of this class of nanoparticles.

## Figures and Tables

**Table 1:** Dimensions and lattice parameters of ZnF particles.

Particles	D_TEM_ (nm)	D_XRD_ (nm)	a (Å)
Zn01	27.33 ± 0.59	14.94 ± 1.12	8.393
Zn02	14.92 ± 0.14	13.85 ± 1.23	8.401
Zn04	13.94 ± 0.13	11.87 ± 1.52	8.418
Zn06	12.87 ± 0.14	11.41 ± 1.34	8.435
Zn08	11.47 ± 0.1	10.98 ± 0.98	8.438
Zn10	10.78 ± 0.09	10.34 ± 0.95	8.444

**Table 2:** Magnetic hysteresis parameters of Fe_3_O_4_ and ZnF particles at room temperature.

Sample	M_s_ (emu/g)	H_c_ (mT)	M_r_ (emu/g)
Fe_3_O_4_	83	22	22.5
ZnF01	90	18	21.5
ZnF02	80	16	17
ZnF04	56	14	9.5
ZnF06	38	13	6.1
ZnF08	23	11	2.4
ZnF10	13	10	1.3

## Data Availability

All data available are reported in the article and the Supplementary Materials.

## Supplementary Materials

Supplementary Materials can be found at https://www.mdpi.com/article/10.3390/pharmaceutics13122148/s1. Figure S1: Large scale TEM images of ZnF particles, Figure S2: Iron and zinc percentages and theoretical and experimental evaluated zinc doping level in all six ZnF samples, Figure S3. The hydrodynamic diameter of all six types of ZnF particles dispersed in water, Figure S4: Heating curves and their corresponding temperature change ΔT versus time curves fitted with Box-Lucas equation, Figure S5: Calibration curve for Fe^3+^ quantification by the Leibig reaction, Figure S6: Heating curves of ZnF01 and ZnF02 particles internalized in A549 and BJ cells, Figure S7: Saturation temperatures of ZnF01 and ZnF02 particles internalized in A549 and BJ cells, Table S1: Values of iron and zinc percentages and zinc doping level in ZnF samples, Table S2. Mean hydrodynamic diameter and polydispersity index values, Table S3: Fitting parameters of SAR evolution with H.
