# Supplementary material for: The Effect of Zn-Substitution on the Morphological, Magnetic, Cytotoxic, and In Vitro Hyperthermia Properties of Polyhedral Ferrite Magnetic Nanoparticles
Source: Pharmaceutics. 2021 Dec 14;13(12):2148. doi: 10.3390/pharmaceutics13122148 (PMC8708233; doi:10.3390/pharmaceutics13122148)
Supplement: Supplementary file 1 [file pharmaceutics-13-02148-s001.zip › pharmaceutics-1481345-supplementary.pdf]

pharmaceutics

Supplementary Materials: The Effect of Zn-Substitution on the Morphological, Magnetic, Cytotoxic, and In Vitro Hyperthermia Properties of Polyhedral Ferrite Magnetic Nanoparticles

Ionel Fizesan , Cristian Iacovita, Anca Pop, Bela Kiss, Roxana Dudric, Rares Stiufiuc, Constantin Mihai Lucaciu and Felicia Loghin

1. Large Scale Transmission Electron Microscopy Analysis

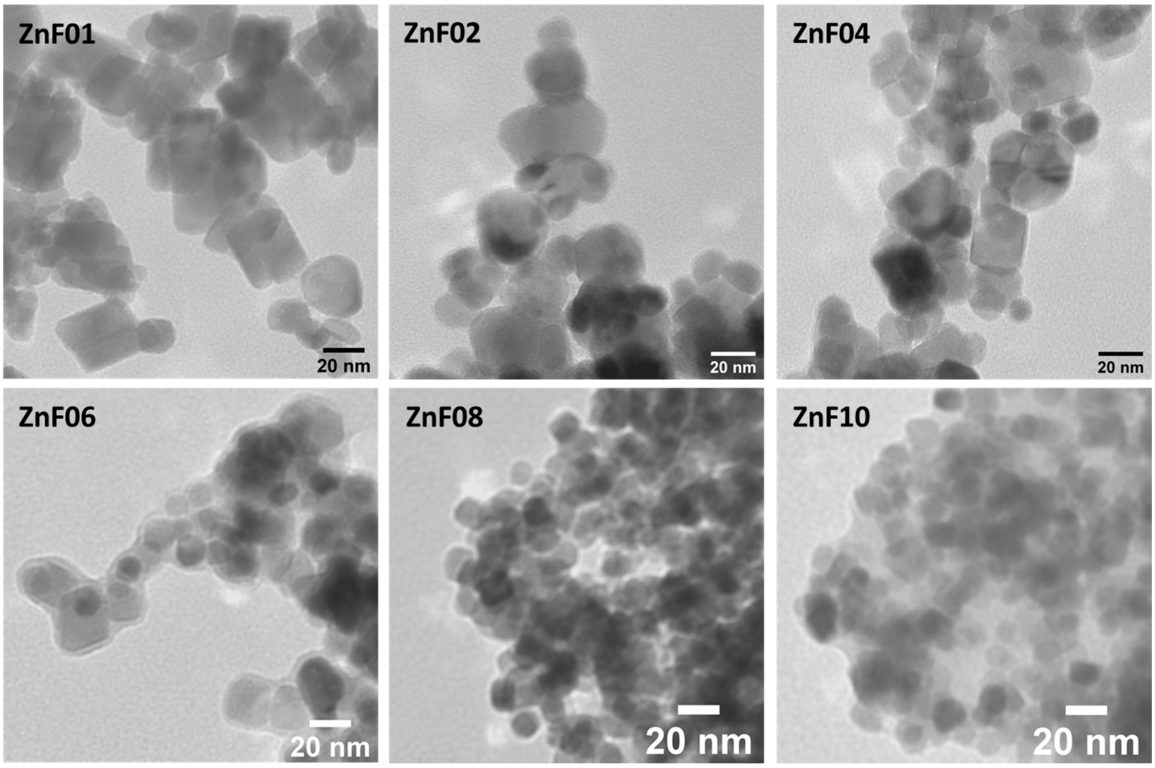

Figure S1. Large scaleTEM images of ZnF particles showing the PEG layer around particles. For the first three types of particles, the PEG layer is very thin, it is thicker and visible for ZnF06 particles, while the particles from samples ZnF08 and ZnF10 are embedded in the PEG carpet.

2. Evaluation of Zinc doping Level in ZnF Samples

Firstly, the ZnF particles were measured gravimetrically after solvent removal in a dry oven at 60 °C for 24 h until the stable mass of 5 mg was reached. Afterward, the iron concentration was measured using the Leibig assay from all samples containing the same mass. We observed that the % iron from the total ZnF particle mass decreased from 60% to 38% with increasing the Zn precursor amount in the mixture (Table S1). If it is assumed that the coating layer represents 10% to 15% from the total mass of particles used in determination, the amount of iron with respect to a pure (confirmed by XRD) and uncoated spinel structure decrease from 67.8% to 42.94% with increasing the Zn precursor amount in the reaction mixture (Table S1 and Figure S1a). Pure spinel structure contains 73% metal ions and 27% oxygen. Therefore, the difference up to 73% is given by the incorporated Zn, which increases from 5.2% to 30.06% when going from ZnF01 to ZnF10 samples (Table S1

and Figure S1a). The Zn doping level, which means the ″x″ in the formula $Zn_xFe_{3-x}O_4$, varies from 0.21 (±0.06) for ZnF01 particles to 1.23 (±0.04) for Zn10 particles (Table S1 and Figure S1b). Concerning the theoretical ″x″, values, the experimental ones are lower (Figure S1b). The difference became larger as the Fe/Zn molar ratio is increased in the reaction mixture.

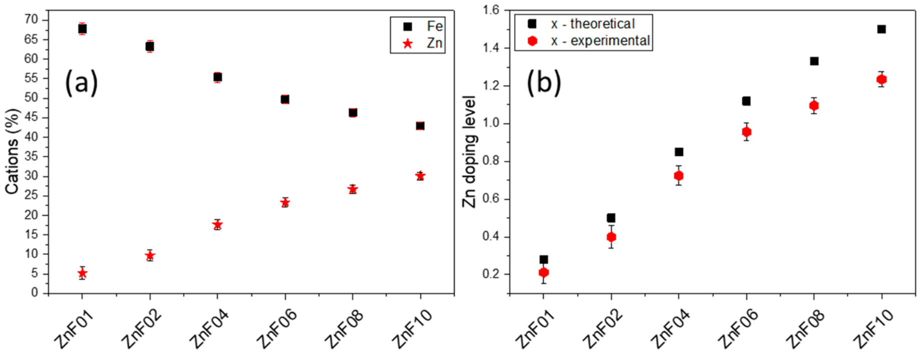

Figure S2. (**a**) Iron and zinc percentages in all six ZnF samples. (**b**) Theoretical and experimental evaluated zinc doping levels in all six ZnF samples.

Table S1. Values of iron and zinc percentages and zinc doping level in ZnF samples.

Sample	Iron (%)	Iron Upon Coating Correction (%)	Zinc (%)	Theoretical x	Experimental x
ZnF01	60	67.8 ± 1.55	5.2 ± 1.55	0.28	0.21 ± 0.060
ZnF02	56	63.28 ± 1.45	9.72 ± 1.45	0.5	0.39 ± 0.059
ZnF04	49	55.37 ± 1.26	17.63 ± 1.26	0.85	0.72 ± 0.052
ZnF06	44	49.72 ± 1.13	23.28 ± 1.13	1.12	0.95 ± 0.046
ZnF08	41	46.33 ± 1.06	26.67 ± 1.06	1.33	1.09 ± 0.043
ZnF10	38	42.94 ± 0.98	30.06 ± 0.98	1.5	1.23 ± 0.040

3. Dynamic Light Scattering Analysis

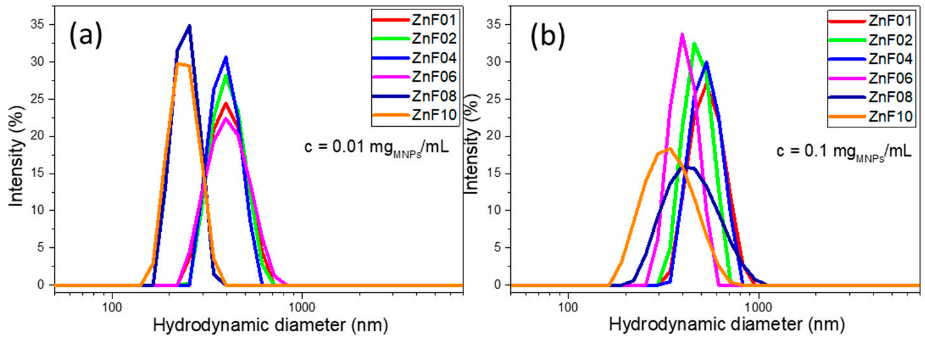

Figure S3. Hydrodynamic diameter resulted from DLS measurements of all six types of ZnF particles dispersed in water at concentration of (**a**) 0.01 mg_{MNPs}/mL and (**b**) 0.1 mg_{MNPs}/mL.

Table S2. Mean hydrodynamic diameter and polidispersivity index values obtained from DLS data.

Sample	c = 0.01 mg_{MNPs}/mL		c = 0.1 mg_{MNPs}/mL	
	Mean Hydrodynamic Diameter (nm)	Polydispersity Index	Mean Iydrodynamic Iiameter (nm)	Polydispersity Index
ZnF01	395	0.34	520	0.03

ZnF02	395	0.36	490	0.04
ZnF04	395	0.28	510	0.03
ZnF06	395	0.29	395	0.27
ZnF08	245	0.27	425	0.54
ZnF10	240	0.17	335	0.41

4. Heating Curves of ZnF01, ZF02, ZnF03, and Znf04 Particles Dispersed in Water at Two Different Concentrations of Iron Content

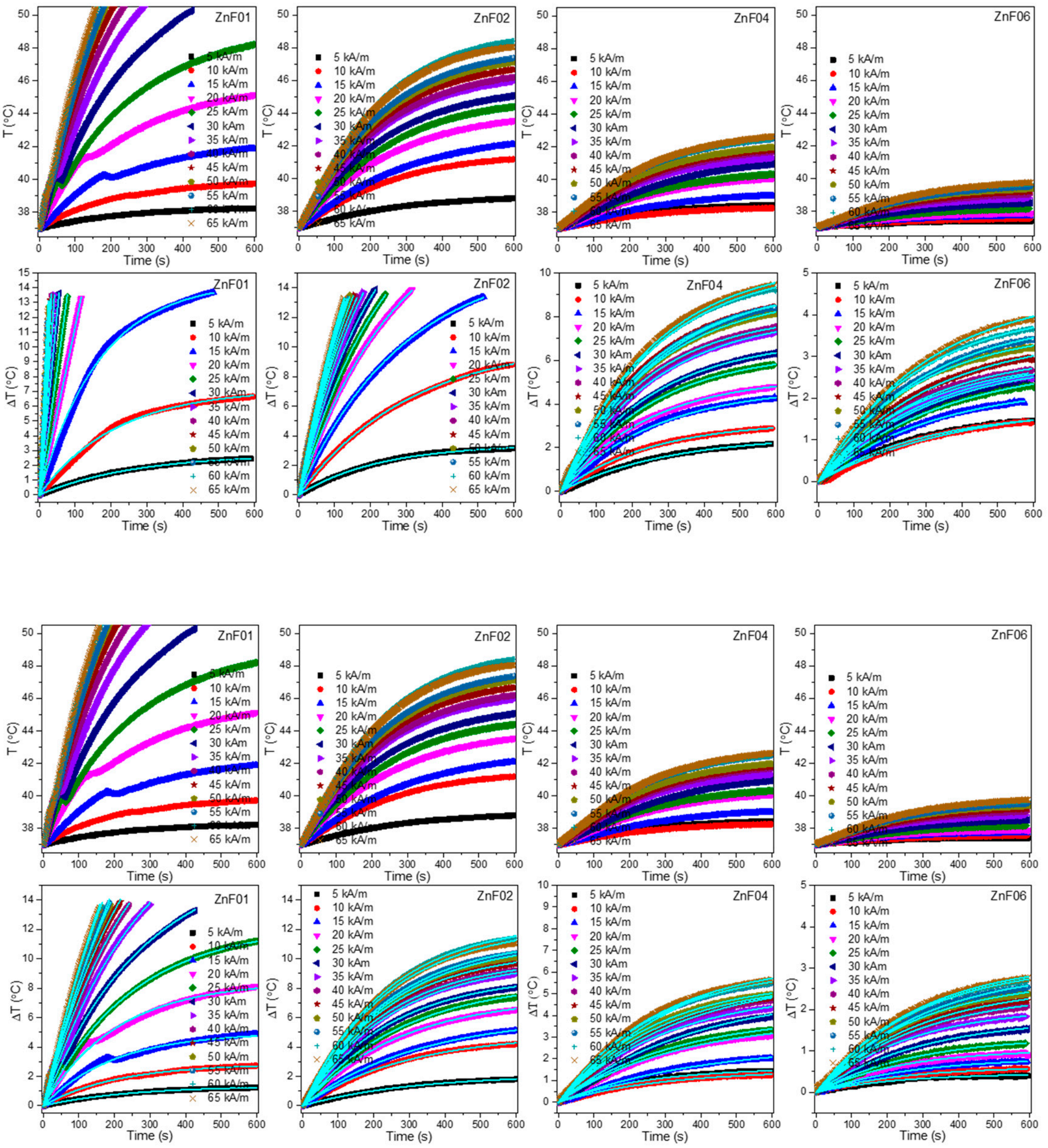

Figure S4. Groups of panels displaying the heating curves and their corresponding temperature change ΔT versus time curves fitted with Box-Lucas equation (blue curves) of ZnF01, ZF02, ZnF03, and ZnF04 particles dispersed in water at concentrations of 4.0 mg $_{Fe}$/mL (first two rows) and 1.0 mg$_{Fe}$/mL (the last two rows) recorded as a function of AC magnetic field amplitudes at a frequency of 355 kHz.

5. The Method of Specific Absorption Rate (SAR) Determination

The specific absorption rate (SAR) is defined as the heat released from a suspension of MNPs in unit time reported to the mass of iron content. It was used to quantify the heat performance of MNPs. For reliable determination of SAR, the temperature change ΔT versus time curves - where $\Delta T = T(t) - T_0$; T(t) is the temperature at time t and $T_0 = 37$ °C, have been fitted with the Box-Lucas equation:

$$\Delta T = \frac{S_m}{k}\left(1 - e^{-k(t-t_0)}\right) \quad (1)$$

where the fitting parameters S_m and k are the initial slope of the heating curve and the constant describing the cooling rate, respectively. Thus, SAR can be calculated as:

$$\text{SAR} = \frac{c\, m\, S_m}{m_{Fe}} \quad (2)$$

where c is the specific heat of the colloid (in our case was approximated with the specific heat of water: $c = 4186.8 \frac{\text{J}}{\text{kg K}}$ the MNPs contribution to the specific heat being negligible), $\text{m} = \rho\text{V}$ is the mass of colloid, taken as the product between the density ($\rho_{water} = 0{,}997 \frac{\text{g}}{cm^3}$)and the volume. The iron concentration of samples was determined using the thiocyanate assay. Before each measurement, the samples have been sonicated for 15 seconds to assure a good colloidal dispersion over the entire aqueous volume. Each SAR value is a mean of three measurements realized on three different samples.

6. Iron Concentration Determination

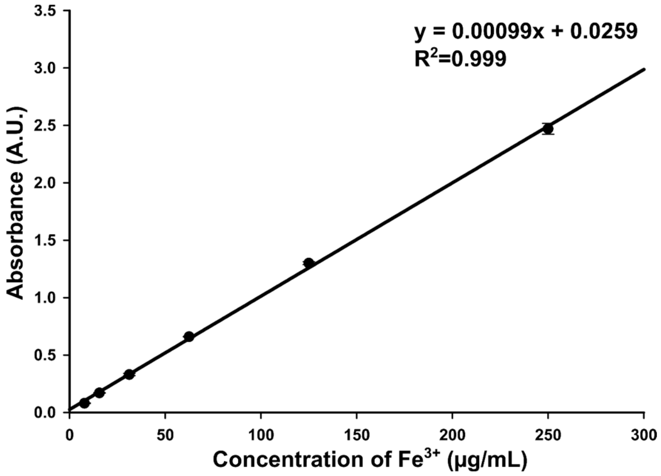

Figure S5. The absorbance of six standard Fe^{3+} colloidal solutions as a function of Fe^{3+} concentration measured at a λ = 490 nm. The values are expressed as mean ± SD of three replicates. The black line represents a linear regression of the experimental values.

7. The Logistic Function Used in Fitting the SAR Values as a Function of H

The sigmoidal evolution of our experimental SAR data with H was well fitted ($R^2 > 0.999$) phenomenologically with a simple logistic function:

$$SAR = SAR_{max}\frac{\left(\frac{H}{H_{cHyp}}\right)^n * \propto}{1 + \left(\frac{H}{H_{cHyp}}\right)^n * \propto} \quad (3)$$

with:

$$\propto = \frac{n+1}{n-1} \quad (4)$$

where SAR_{max} - the saturation value of the SAR, H_{cHyp} - the hyperthermia coercive field, the value of the H for which the function presents the highest slope or the H at which the first derivative of SAR against H presents a maximum, and the exponent n – which indicates how steep is the dependence of SAR on H. The values of these parameters for all four types of MNPs at each iron concentration are provided in Table SI1.

Table S3. Fitting parameters of SAR evolution with H.

Sample	Conditions	c (mg_{Fe}/mL)	SAR_{max} (W/g_{Fe})	H_{cHyp} (kA/m)	Power Coefficient n
ZnF01	Water	4.0	525 ± 22	22.42 ± 1.4	2.53 ± 0.18
		1.0	820 ± 50	21.98 ± 2.9	1.83 ± 0.01
ZnF02	Water	4.0	180 ± 4	23.46 ± 0.8	1.59 ± 0.05
		1.0	325 ± 37	35.05 ± 1.5	1.14 ± 0.11

8. Heating Curves of ZnF01 and ZnF02 Internalized in A549 and BJ Cells at Different Concentration

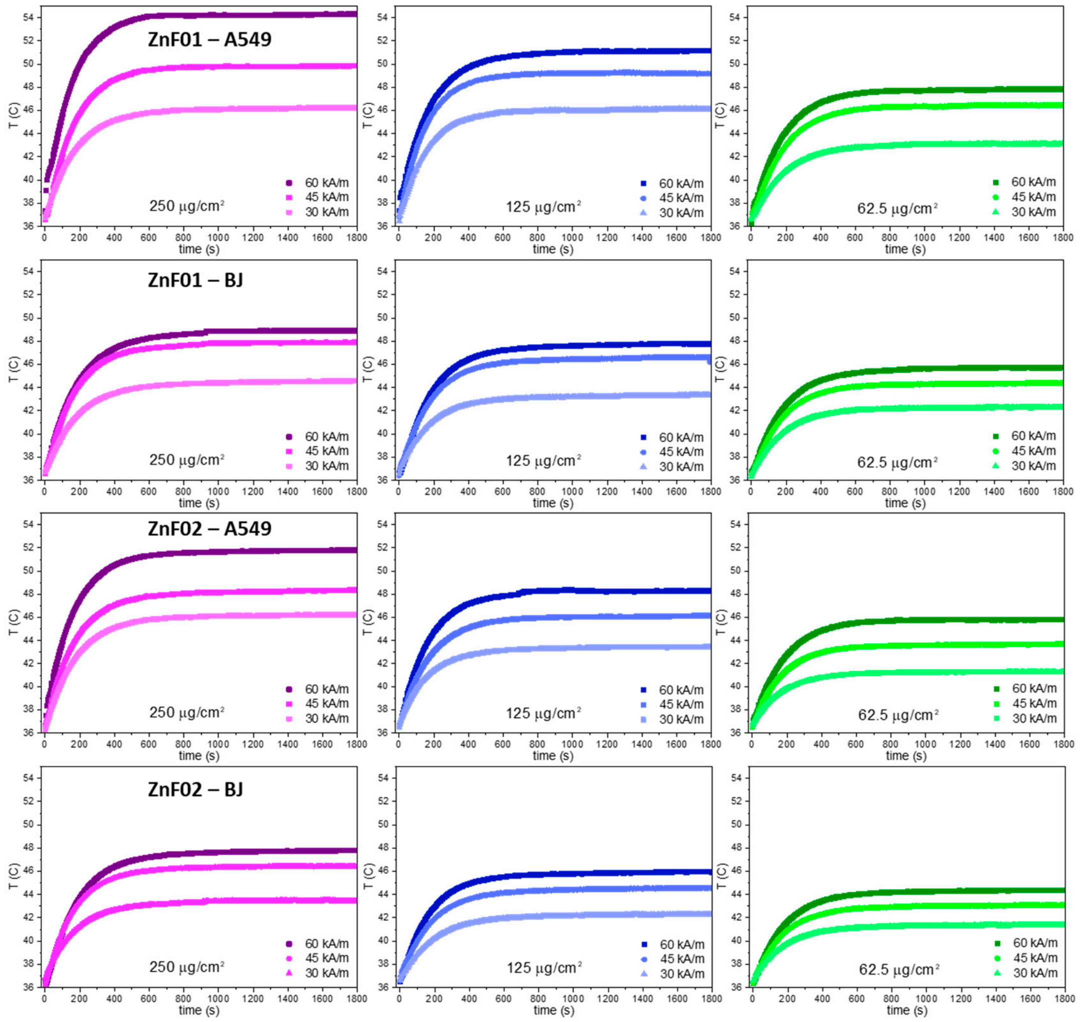

Figure S6. Groups of panels displaying the heating curves of ZnF01 and ZnF02 particles internalized in A549 and BJ cells at different concentrations, recorded at three different H values of 30 kA/m, 45 kA/m, and 60 kA/m and a constant frequency of 355 kHz.

9. Saturation Temperatures Reached during In Vitro MH Experiments

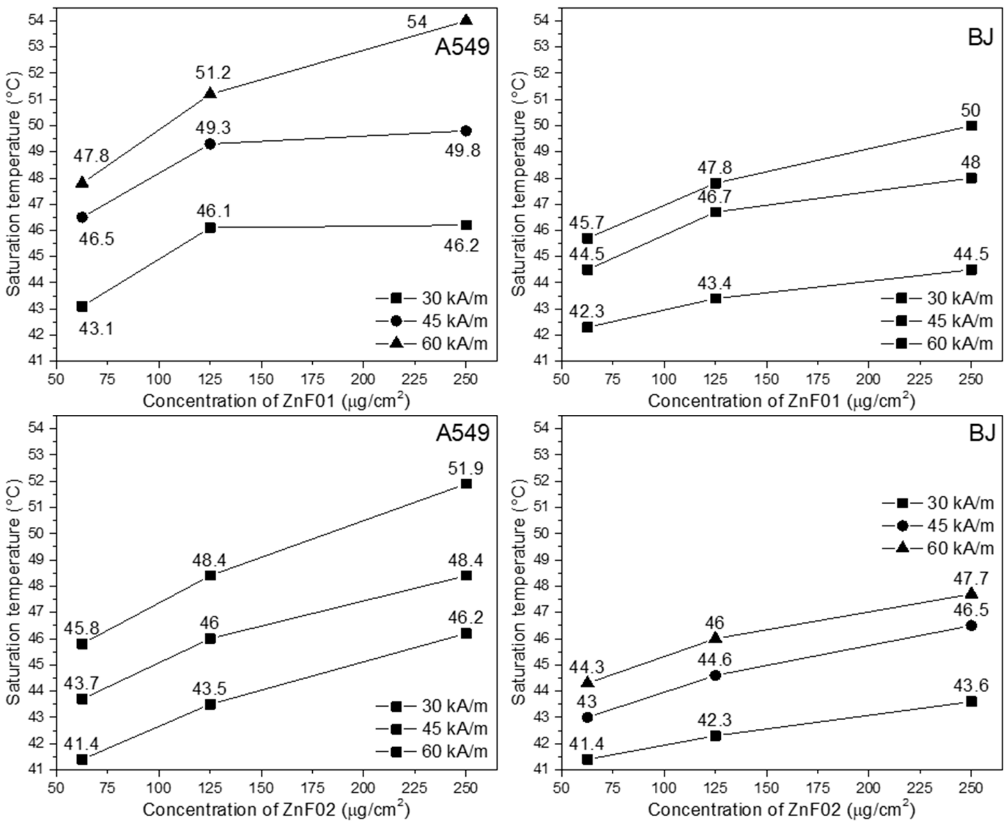

Figure S7. Saturation temperatures of ZnF01 and ZnF02 particles internalized in A549 and BJ cells at different concentrations reached upon 30 minutes exposure to AC magnetic field of three H values (30, 45, and 60 kA/m) and at a frequency of 355 kHz.
